# Dielectric Nanorod Scattering and its Influence on Material Interfaces

**DOI:** 10.1038/s41598-017-03721-w

**Published:** 2017-06-27

**Authors:** Gauri M. Mangalgiri, Phillip Manley, Wiebke Riedel, Martina Schmid

**Affiliations:** 1 Nanooptische Konzepte für die PV, Helmholtz Zentrum Berlin für Materialien und Energie, 14109 Berlin, Germany; 2 Freie Universität Berlin, Department of Chemistry, 14195 Berlin, Germany; 3 Freie Universität Berlin, Department of Physics, 14195 Berlin, Germany

## Abstract

This work elaborates on the high scattering which dielectric nanorods exhibit and how it can be exploited to control light propagation across material interfaces. A detailed overview of how dielectric nanorods interact with light through a combination of dipolar scattering and leaky modes is performed via outward power flux calculations. We establish and account for design parameters that best result in light magnification owing to resonant behavior of nanorods. Impact of material parameters on scattering and their dispersion have been calculated to establish that low loss dielectric oxides like ZnO when nanostructured show excellent antenna like resonances which can be used to control light coupling and propagation. Interfacial scattering calculations demonstrate the high forward directivity of nanorods for various dielectric interfaces. A systematic analysis for different configurations of single and periodic nanorods on air dielectric interface emphasizes the light coupling tendencies exhibited by nanorods to and from a dielectric. Spatial characteristics of the localized field enhancement of the nanorod array on an air dielectric interface show focusing attributes of the nanorod array. We give a detailed account to tailor and selectively increase light propagation across an interface with good spectral and spatial control.

## Introduction

Engineering dielectric materials to subwavelength structures has triggered recent interest in order to achieve control over light enhancement and manipulation^1–3^. Dielectric materials have been preferentially selected over their metallic counterparts as a result of their established superiority in terms of the absence of parasitic absorption as well as increased stability^3–9^. Moreover there is a large selection of dielectric materials available in terms of desired dispersive properties. Nanostructuring a material where effects of light enhancement could be exploited is one of the approaches to acquire desired control over light propagation and local confinement. III-V materials fabricated into nanostructures have been in focus in order to enhance light collection and dissemination in optoelectronic devices such as LEDs and solar cells^10–12^. It has been proved that nanowire solar cells have an higher efficiency as compared to their planar counterparts. Recent attempts have also been in the direction of studying the light propagation in relatively short length (<10 µm) nanowires in order to enhance absorption in solar cells. The said work also establishes similarities between Mie resonances for cylinders^13^. The major focus is to harness the waveguide modes present in nanowires which help in confining light for enhancing absorption. Links have also been established between the absorption of a single nanowire and the absorption of a nanowire solar cell array made up of III-V materials such as InP^14–16^. These investigations establish the advantages of a nanowire geometry in terms of light confinement and interaction inside the nanowire. Nanostructuring absorber materials therefore helps in improved light guiding into the absorber material increasing conversion efficiencies. Another approach is to strategically design low loss dielectric nanostructures and integrate them with materials which require light enhancement. Integration of nanostructured dielectrics with absorbers for energy conversion helps in achieving higher efficiencies due to light localization of the resonant nanostructures.

Enabling design induced optical resonances in nanostructures results in external light radiation as exhibited by antennas^17–22^. Light enhancement in the vicinity of the structures is the principal advantage of this design where the nanostructures are deployed in absorbers in which absorption enhancement is desired^20^. Spherical dielectric nanoparticles have been of interest due to Mie resonances which they exhibit and have proven to increase light control and localization upon integration with optoelectronic devices^23^. The limitation of these small particles is that they do not offer strong directivity^24–27^. Additionally, efforts have been made to examine the light scattering properties of small silicon cylindrical particles by systematically observing their resonances for different background conditions. These small cylindrical particles show scattering resonances via magnetic and electric modes which result in localised light enhancement^28, 29^. The gap between small cylindrical particles and long nanowires made from low loss dielectric materials comprises of cylindrical nanocolumns and rods which have been looked at experimentally and have shown increase in the optoelectronic performance in the surrounding environment^30^. In addition to providing localised light enhancement and scattering benefits such nanocolumns and rods would also increase directivity, thereby control propagation across an interface. Materials typically used in this configuration are transparent conductive oxides which help providing localised light enhancement in solar cells as well as function as electric contacts^17, 31^. Most of these oxides have a low loss and a relatively high refractive index. This opens a window for design and implementation for such low loss structures to direct and manipulate light as desired^17^. It is however important in order to have an elaborate understanding of how exactly light interacts with such nanorods and establish a link between the governing parameters. This would aid in designing nanorod based structures that help in controlling light across a given set of interface optical parameters for different optoelectronic devices.

**Figure 1:**
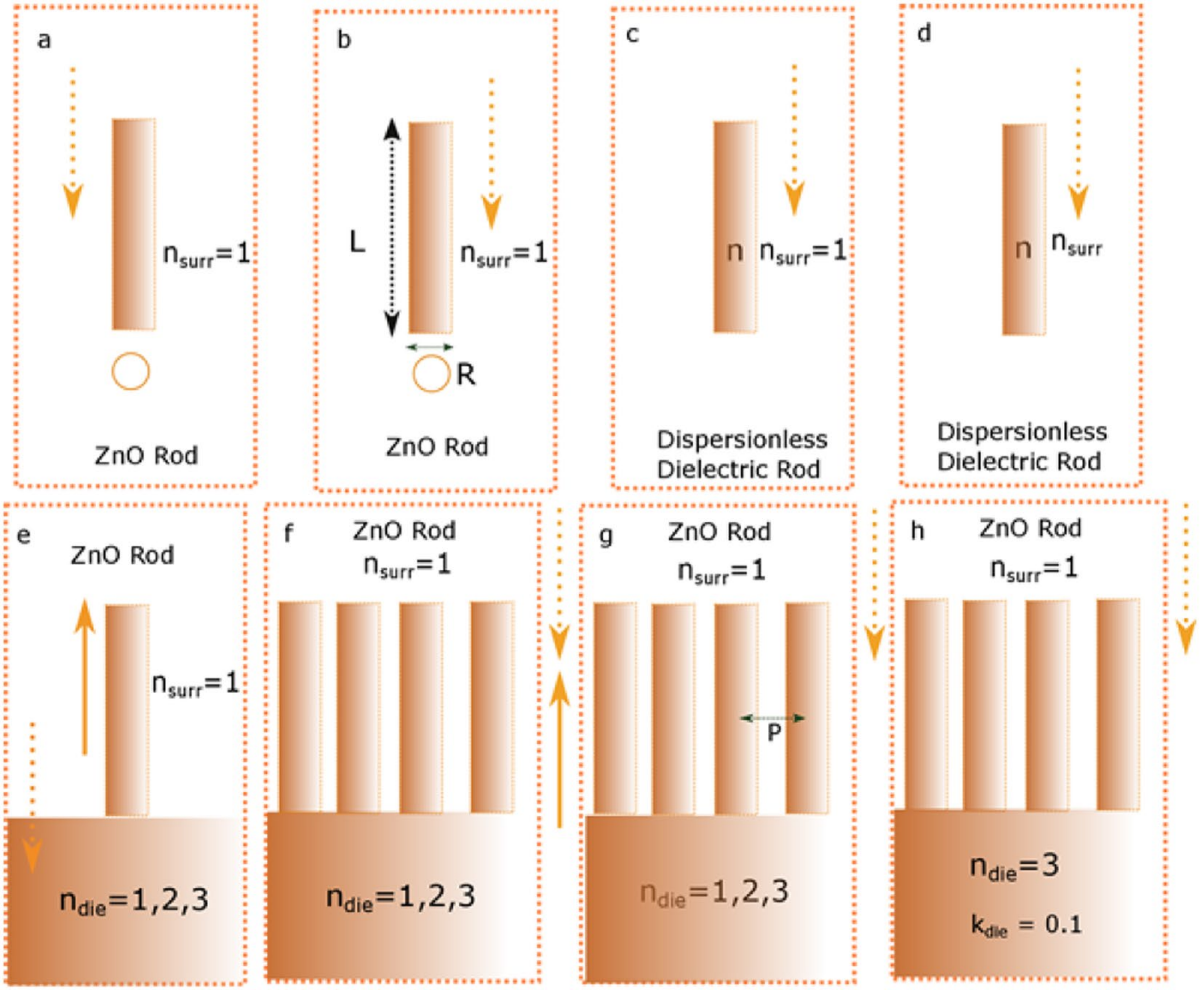
Overview of various configurations of ZnO and dielectric nanorods investigated for in homogenous dielectric and across air dielectric interfaces in the order as examined in this paper.

In our work, we analyze with the aid of systematic simulations, light propagation properties of dielectric nanorods and establish design parameters for utilizing them for light control. Figure 1 gives an overview of the investigated nanorod configurations and the physical properties and their extent of homogeneity for the surroundings. Furthermore, the direction of light incidence with respect to the interface is depicted. An investigation of how a dielectric nanorod interacts with light for a suitable dielectric material like ZnO is performed first (Fig. 1(a)). This is followed by further investigations on how geometry (radius R and length L) influences scattering, Fig. 1(b). In order to understand how this scattering spectra is governed by material optical data scattering calculations for dispersion less dielectric rods (refractive index n + i*k) of the same geometry are performed and correlated to the results with that of a ZnO rod, Fig. 1(c). Subsequently we investigate how these properties change when the refractive index of the surrounding material (n_surr_) is changed from air to a dielectric, Fig. 1(d).

Since most optoelectronic applications including and especially solar cells inevitably contain an interface to a dielectric (n_die_), we investigate how this dielectric interface influences nanorod scattering properties as well as how nanorods influence light propagation across the interface, (dielectric refractive index n_die_, Fig. 1(e)). Axial light incidence is considered in all cases to investigate electric field strength variance across the rod length. Links between scattering and enhancement properties of a single nanorod and that of a cartesian nanorod array, are established on different air dielectric interfaces for a lossless dielectric (Fig. 1(f)). Our idea is to analyze how to preferentially guide light over a given interface and spectrally control it with the help of nanorod arrays which exploit the scattering and light enhancement of single dielectric nanorods. We investigate how different pitches (P, Fig. 1(g)) spectrally influence interface properties such as reflection and transmission via different air-dielectric interfaces. Finally we look at how nanorod arrays enhance absorption in the dielectric (with absorption coefficient k_die_) for a given air-dielectric interface (Fig. 1(h)). A detailed description of how nanorod arrays result in increased near field in absorbing materials is shown at the end of the paper.

## Results and Discussion

### Overview of Single ZnO Nanorod Scattering

**Figure 2:**
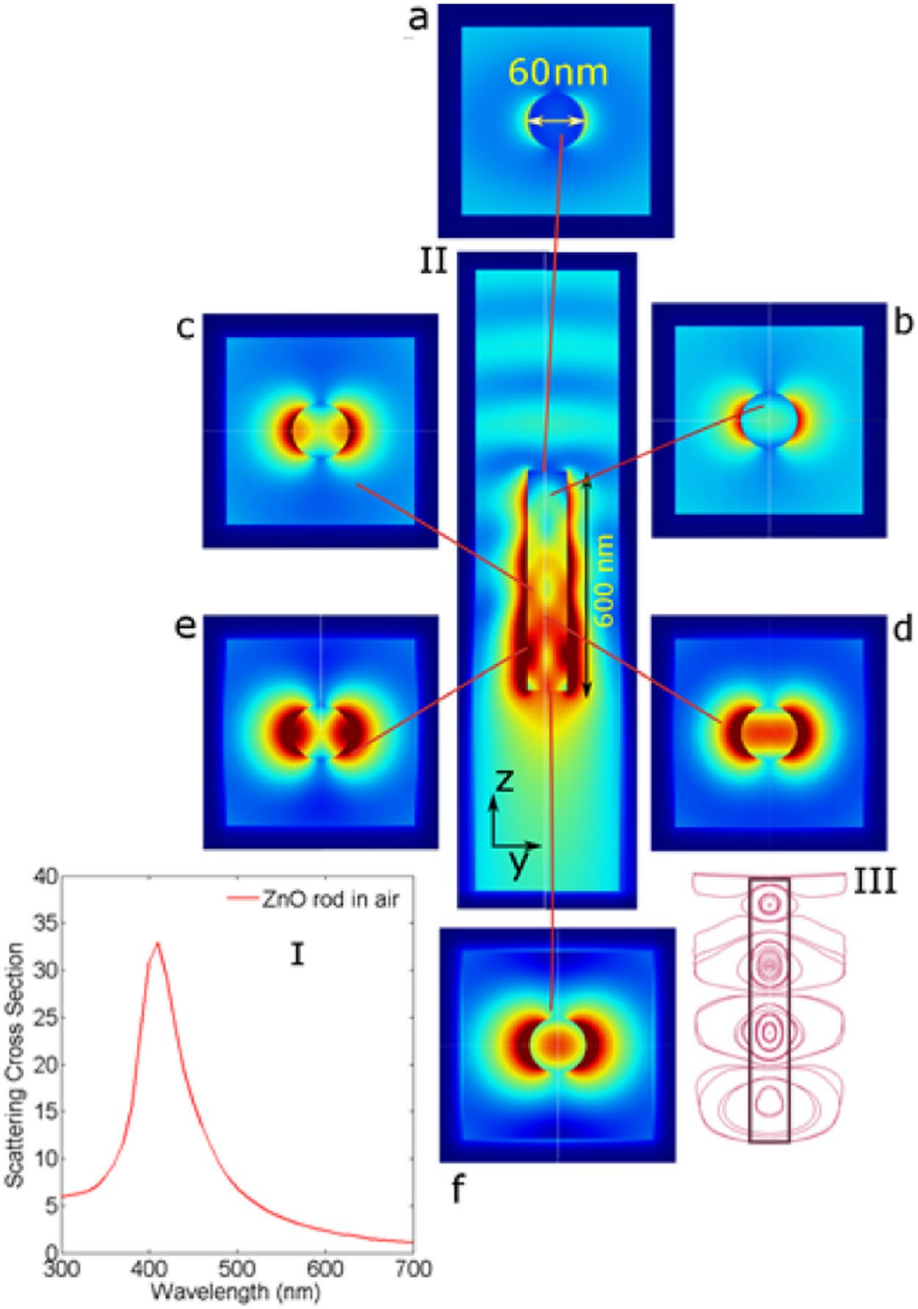
(I) Scattering cross section of a ZnO rod in air. (II) Longitudinal electric field plot of ZnO nanorod in air at the wavelength of 410 nm. (**a–f**) Transverse sections of the rod as light propagates along the rod length showing evolution of electric field inside the rod. (III) Streamline plot of the electric field indicating expansion of electric field as the light propagates along the rod.

We start with the evaluation of scattering cross-section of a ZnO nanorod in air with radius 60 nm and length 600 nm. Light is axially incident on the rod with the electric field polarized perpendicular to the rod axis. The material optical data is retrieved from reflection-transmission measurements of a planar layer of ZnO using transfer matrix method^32, 33^. Figure 2(I) shows the scattering cross section of the nanorod in air with a characteristic resonance at 410 nm. With the aid of near fields as observed along the length of the rod we analyze the light flow pattern resulting in the spectral trend of the scattering cross section, (Fig. 2(II), and at the various lateral sections (xy-plane)). Figure 2(a) shows the field distribution at the face where light is first incident. Two dipole lobes in the rod exterior are present. Moving along the rod length we see a central field maxima emerging in Fig. 2(b). The field maxima in the interior corresponds to the field confined by the rod. This confined field by the rod is a function of its material and geometry indicating a waveguide like behavior. Figure 2(c) highlights that the mode in the rod interior changes from being centralized to being confined to the rod edges. In Fig. 2(d) we observe outward propagation towards along the edge of the rod. Figure 2(e) shows increased intensity along the edge interior as well as in the exterior dipoles. Figure 2(f) finally shows the expansion of the exterior modes which result in high field intensity at the rod end. The streamline plot in Fig. 2(III) illustrates further the outward expanding interior fields along the rod length. It also highlights that the longitudinal (yz) field is not purely oscillatory as in the case of a conventional waveguide owing to the rod air interface. This deviation is caused by the light scattering which occurs at the end facets of the rod. The superposition of these two effects gives rise to the characteristic field distribution in the rod. The electric field in the interior expands in the outward direction Fig. 2(II) shows field intensity maxima alternate between the center and the rod edges as light propagates across the rod length. Across the length, both the edge and the central field maxima become stronger in intensity in the direction of propagation. This can be explained by the fact that the electric field moves radially outward. Figure 2(a–f) moreover indicate how the field in the rod exterior is enhanced by the externally leaking interior field. As more of the field couples to the exterior, the area enclosed by the streamlines increases. At the bottom edge where the light leaves the rod, we see that the lobe expands which escapes the rod resulting in high intensity near the rod edge. The near field images showing the rod’s transverse view indicate the presence of electric dipoles at the rod surface along the length of the rod which radiate light into the far field. These dipole sources arise due to the response of the microscopic dipoles being polarized by the incident field which confirms dielectric scattering.

These dipoles are modulated in intensity by the presence of a mode propagating internally along the rod length. The transverse field diagrams plotted along the rod length indicate how the intensity of these dipoles are modulated along the rod. The resultant power outflow due to this phenomenon is used to calculate scattering. We use the surface integral of the Poynting vector flux normal to the domain boundary to evaluate scattering^34^. The ratio of Poynting vector integrals on the computational domain to the input power normalized to the cross sectional area of the cylinder with respect to the incident light gives the evaluated scattering cross section in the Fig. 2(I) as well as wherever described in the subsequent text. The figure shows the scattering cross section of a single nanorod in air which has a single peak at 410 nm along with a maximum value of 30. A scattering cross section of 30 implies that light effectively sees an area corresponding to 30 times the actual geometric cross section. At this scattering resonance, since the material optical absorption is minimum, light propagating through the rods gains amplitude across the length of the rod which justifies the increasing field strength along the propagation axis. In the transverse plane, the y-component of the E-field spreads outwards with increasing magnitude along the length of the rod, confirming the presence of a leaky waveguide mode (supplementary information). The field in the interior thereby couples to the exterior via leaky waveguide modes. Nanorods therefore exhibit leaky waveguide mode enhanced scattering^35^. These fields leaking in the exterior of the rod are quantified by the Poynting flux components which give rise to a high scattering resonance.

### Influence of Geometry on ZnO Nanorod Scattering

**Figure 3:**
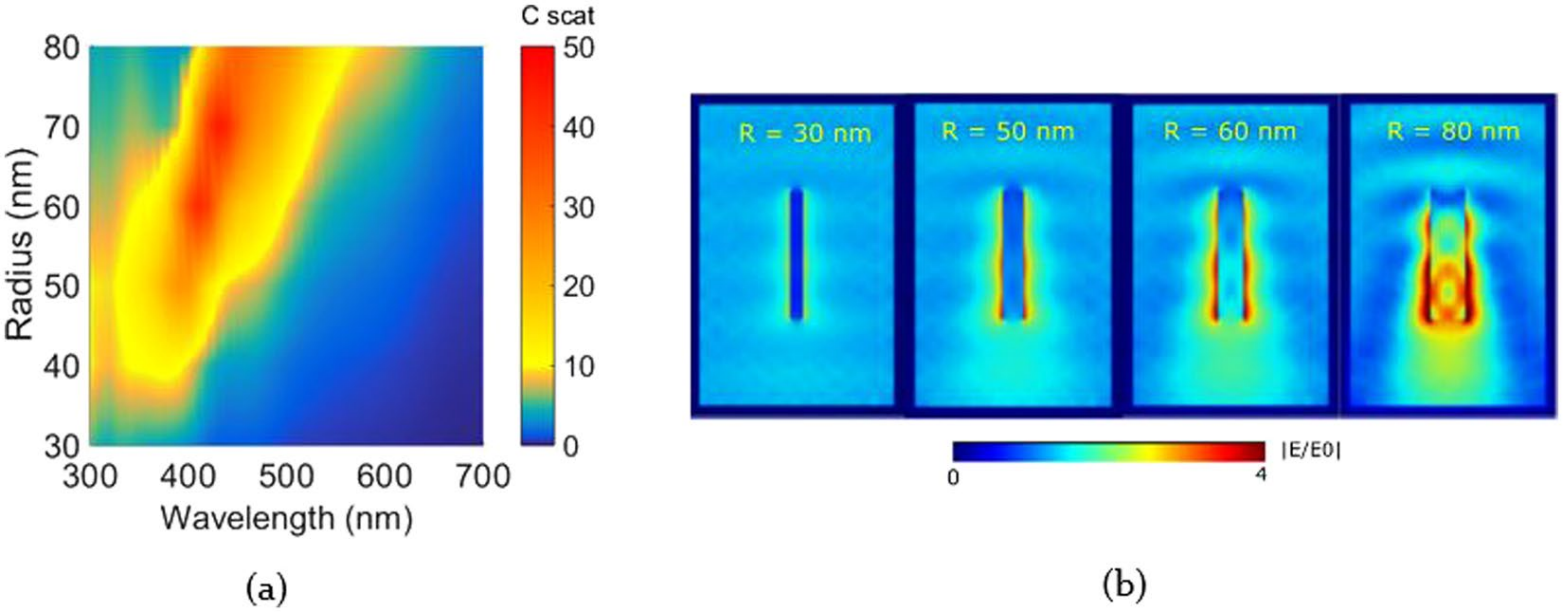
(**a**) Scattering cross section spectra of a 600 nm long ZnO nanorod in air calculated for different radii (**b**) near field observed at light wavelength of 500 nm for radii 30, 50, 60 and 80 nm.

For rods made up of ZnO we firstly investigate the influence of radius on the rod scattering and maintain the length at 600 nm. The radius is swept between 30 and 80 nm to focus on moderate aspect ratios. The incident light is parallel to the cylinder axis from the top and the electric field is polarized perpendicular to the rod axis. Figure 3(a) illustrates scattering as a function of radius and wavelength evaluated from the above calculation. Figure 3(b) shows the near field distributions for radii R = 30, 50, 60 and 80 nm, going from left to right, at a wavelength of 500 nm in the centre of the scattering diagram.

It is observed that increasing the rod radius red shifts the scattering peak. The fundamental resonant mode at which field is initially confined to the rod interior shifts to a higher wavelength. Dielectric waveguides show a similar red shift in the cut off frequency by increasing radius^36^. There is a specific wavelength and radius range where the scattering is extremely high. This range corresponds to the region where we have pronounced leaky waveguide modes which enhance scattering. In the scattering graph this corresponds to the wavelength range between 400–500 nm where nanorods have a radius of 30–80 nm. For all other wavelengths the total scattering is relatively low for rods with radii 50–80 nm and the corresponding near field diagrams show low field confinement inside the rod. The waveguide response of the rod is not matched for the given radius and wavelength in this case. Thereby the response seen is purely due to polarisation of the microscopic dipoles which gives rise to the dipoles in the exterior.

**Figure 4:**
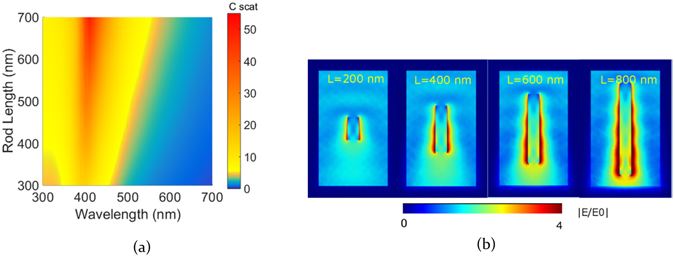
(**a**) Scattering cross section spectra for different ZnO rod lengths having radius 60 nm in air (**b**) near field observed at light wavelength of 500 nm for lengths 200, 400, 600 and 800 nm.

In the next step, the influence of the rod length on the scattering cross section was investigated for a ZnO nanorod in air. We maintain a constant radius of 60 nm for the same material data and the length is swept between 100 and 700 nm. These limits are set in order to facilitate outward propagation of light across the rod edges. The rod essentially acts like poor waveguide leaking out all the light confined by it into its exterior which requires that the aspect ratio isn’t too high for a given radius. It is observed that the wavelength at which a scattering peak is obtained remains constant for changing lengths ﻿(Fig. 4a). It is interesting to note that the scattering amplitude increases with increasing length however the aspect ratio between radius and length would still play a role in determining the extent of paraxial to wide angle outward radiation of the near fields. ﻿An﻿ increase﻿ in length implies more volume of the dielectric material that interacts with light resulting in an increase in total scattering. The adjoining near field figures of different nanorod lengths (200, 400, 600 and 800 nm) show an overall similar behavior of field patterns along the rod length while showing different field maxima at the rear edge of the rod. For the longer rod it is seen that aforementioned field maxima at the interior rod edges propagate across the rod to give rise to a higher field strength at the edge where light leaves the rod﻿.

### Influence of Material Data on Nanorod Scattering

**Figure 5:**
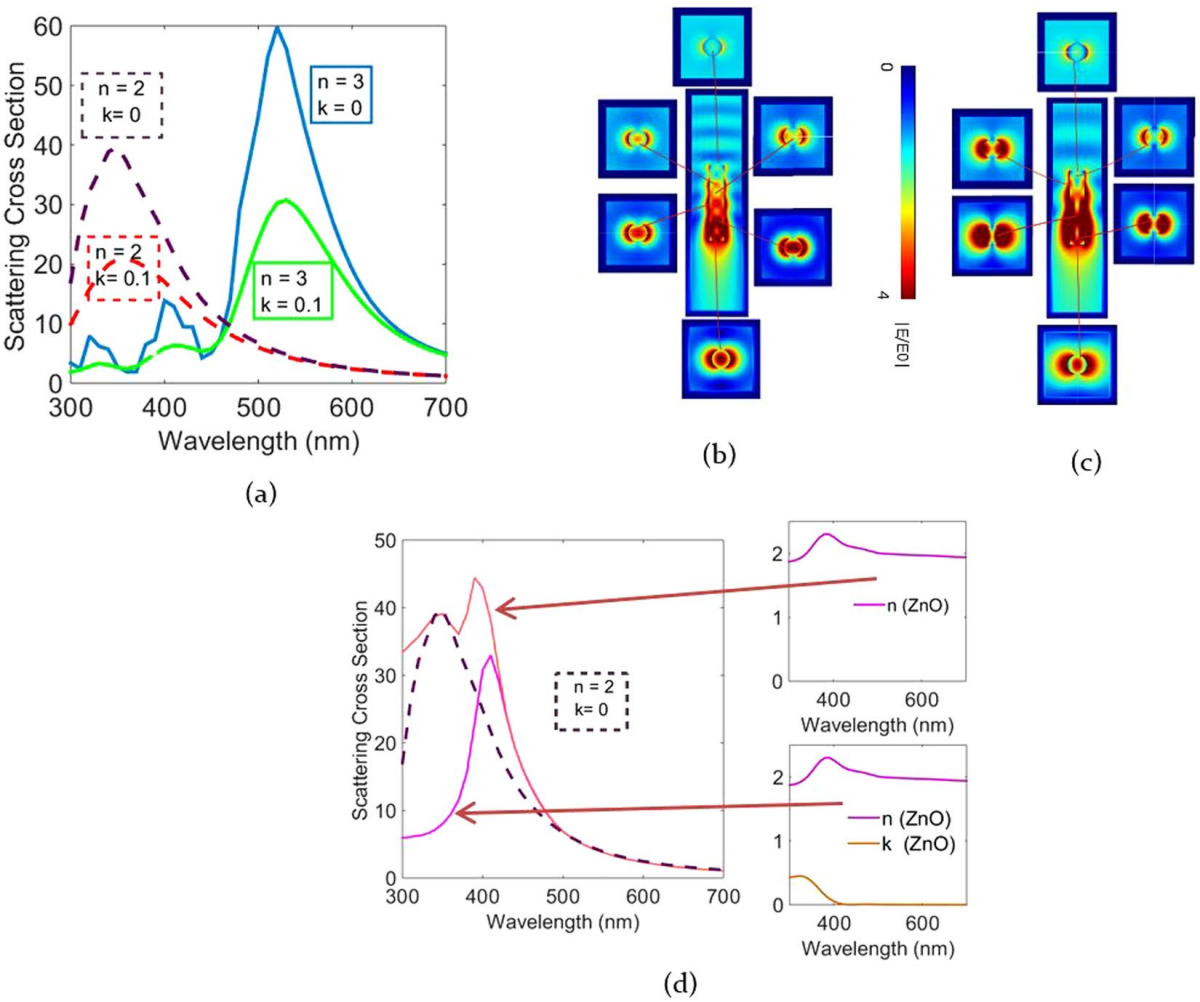
(**a**) Scattering cross section for rod refractive indices n + ik = (2 + 0i, 2 + 0.1i, 3 + 0i, 3 + 0.1i) (**b**) near field images of rod rod with index 2 + 0i at its resonant wavelength (350 nm) (**c**) near field images of a rod with index 3 + 0i at the resonant wavelength (530 nm) (**d**) scattering curves for ZnO with dispersive n-k data with and without absorption.

Figures 3 and 4 indicate scattering as a function of geometry for dispersive optical constants of ZnO. This dispersion in material data also contributes to the scattering behavior. In order to explicitly understand the influence of the material optical data on scattering and to utilize it to maneuver scattering as per requirement, the rod geometry is kept constant while changing the n-k material data values. To study this influence, we maintained a constant geometry, i.e. radius 60 nm and length 600 nm. We then calculate scattering for the following refractive index values n + ik = (2 + 0i, 2 + 0.1i, 3 + 0i, 3 + 0.1i). Figure 5(a) gives an overview of the scattering response for the above set of values and it is evident that the scattering peak largely depends on the refractive index of the rod. It is clearly observed that the scattering peaks red shift with increasing refractive index as seen changing from between n = 2 and n = 3. Additionally, with no absorption, i.e. for k = 0, a higher scattering amplitude is observed. A red shift due to increased refractive index is explained from the fact that a higher refractive index effectively shortens the wavelength inside the material, therefore a higher exterior wavelength is needed in order to obtain the same geometrical resonance inside the rod. Scattering peaks can therefore be either red shifted by increasing the radius or using a high index material. For the higher index rod also higher order modes at the short wavelengths with less amplitude appear. Increasing the refractive index helps in improving the spectral spread of scattering peaks, wherein each of the peaks has a different amplitude.

From the field images in Fig. 5(b), (n = 2,k = 0) and (c), (n = 3,k = 0), the influence of the refractive index on the outward radiation of the internally propagating modes can be seen. It is important to note that the transverse sections are taken at different length positions as against those shown in Fig. 2(a–f). A dispersion free refractive index results in a slightly different light propagation across the rod length resulting in different positions of maxima and minima at the rod center and edge. For the rod with index 2 (Fig. 5(b)) which has a resonance at 350 nm, alternating maxima at the rod center and at the rod edges are observed. For the rod with index 3 (Fig. 5(c)), these maxima are more enhanced (resonant wavelength, 500 nm). Additionally the maxima become wider along the rod length. The positions of the maxima at the edge and the center vary significantly for the two indices and for the higher index, field strength is higher. We then calculate the influence of the material optical absorption coefficient on the rod scattering by performing the calculation with and without optical absorption. The scattering calculations and the underlying n-k values are shown in Fig. 5(d).

The first peak is observed at 350 nm and is seen for both actual dispersive n data (without loss) as well as for the constant n data of n = 2 (repeated in dotted purple lines in Fig. 5(d)). This peak is therefore a geometrical resonance and only depends on the rod radius and the ratio of the rod to exterior refractive index, which in this case is 2. For the dispersive n data another peak corresponding to the maximum value of the n data is seen at 410 nm. The material resonance lies in the full width half maximum of the geometric peak, thereby resulting in a peak of higher amplitude. This peak is independent of the rod radius and is only present for the dispersive n data and not for the constant n data, therefore we may attribute this peak to the rapid change in the n value observed around the band gap of ZnO. If the rod had a dispersion less refractive index which equals to the peak value of ZnO (n = 2.2), the corresponding scattering peak would red shift from 350 nm to 385 nm. This highlights the fact that for the same geometry depending upon the material used the refractive index value can be altered in ordered to change the scattering spectra^37^. When absorption of ZnO is considered the peak at lower wavelengths disappears.﻿

For most common materials a high refractive index is often associated with a high material absorption coefficient which results in most of the light being dissipated into the material^38^. Effective light scattering therefore demands for dielectric materials with high refractive index and low absorption coefficient specific to the application wavelength in consideration via layered structures, doping etc.

### Influence of Surrounding Refractive Index on Nanorod Scattering

**Figure 6:**
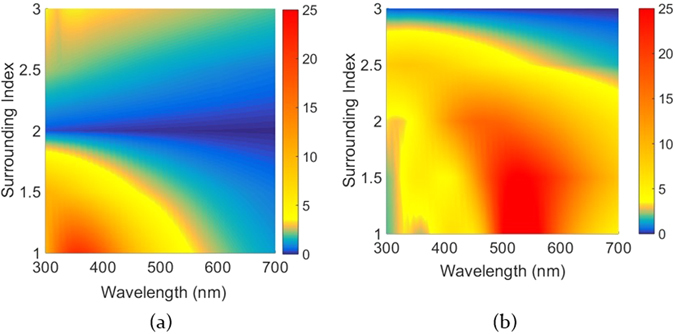
(**a**) Scattering cross section of rod with index (**a**) 2 + 0.1i and (**b**) 3 + 0.1i plotted as a function of varying surrounding medium index and wavelength.

The aforementioned nanorod scattering properties hold true when the nanorod is in air. For optoelectronic applications often the surrounding medium has a different refractive index which will alter the scattering spectra. It is therefore interesting to investigate variations in scattering magnitude and corresponding resonant wavelength when the rod is present in a high index medium. The approach adopted is to vary the refractive index of the surrounding medium between 1 and 3 in steps of 0.5 for a dispersion less rod index of 2 + 0.1i and 3 + 0.1i. The resulting scattering maps plotted against wavelength and surrounding medium index for both rods are shown in Fig. 6(a) and (b) respectively. From the analysis performed the rod with refractive index 2 when present in air has a resonant wavelength as a function of its material and geometry at 350 nm and the effective wavelength inside the rod is 175 nm. For a change of the surrounding, the scattering and the associated wavelength of maximum scattering depends on the rod refractive index relative to the surrounding medium. Starting with air and increasing the refractive index of the surrounding medium up to the rod refractive index, we observe that the scattering peak blue shifts (Fig. 6a). When an electric field is applied to a dielectric medium via an external source, such as incident light, the microscopic dipoles in the bulk of the dielectric are polarized by the applied electric field giving rise to a surface charge. When an electric field is applied across an interface formed by two materials having different permittivities (i.e. different microscopic dipole densities) polarization density in each of the dielectrics changes. This change is as a result of the relative difference of permittivities of the two materials. These two combined effects lead to a polarization surface charge at the interface on the side with the higher permittivity material^39^. When the electric field is directed across material with lower permittivity into the material with higher permittivity the resultant dipole distribution between the two materials is such that a resultant negative surface charge arises in the high permittivity material. Conversely when the electric field is directed from the material with a higher permittivity into the material with a lower permittivity, a positive surface charge arises.

For the nanorods investigated here, the result is always a dipolar source formed due to the incident electric field forming a negative and positive surface charge on either side of the nanorod, depending on the direction of the incident field. When the nanorod has a higher permittivity than its surrounding, the dipole charges are located inside the rod, and act to reduce the field inside the nanorod and enhance it outside (via scattering). Since the surface charge is inside the rod, increasing the medium refractive index will blue shift the resonance wavelength, as a smaller vacuum wavelength outside the rod is needed to reach the same resonance wavelength inside the rod material. When the medium has the higher permittivity the surface charge is located in the medium and acts to increase the field inside the rod. Since the surface charge in this case is outside the rod, increasing the medium refractive index will redshift the resonance, since a larger vacuum wavelength outside the rod is needed to reach the same resonance wavelength in the medium material. Moreover since the polarization intensity depends on the difference between the rod and the surrounding permittivities, for values of refractive index greater than but close to the rod index, the scattering cross section is relatively low. When this difference is high the effective polarization between the rod and the surrounding is high and a high scattering cross section is observed^21, 35^.

For the corresponding rod with higher refractive index value (Fig. 6(b)) a similar trend of red shifting of the scattering spectra is seen. Moreover since the rod has a higher refractive index as compared to the surrounding the scattering value is higher than the one seen in Fig. 6(a). It is therefore verified that for high scattering the difference between the permitttivities of the rod and the surrounding material are significantly large. Moreover it is also important to note that depending upon the refractive index of the surrounding material the nanorod scattering spectra red shifts or blue shifts. This property could be strategically utilized in order to set resonances in different materials and selectively shift spectral response of a particular scatterer. This sets an important criteria of selecting a suitable material where the effects arising out of light enhancement from the rod could be substantially harvested.

### ZnO Nanorod on an Air Dielectric Interface

**Figure 7:**
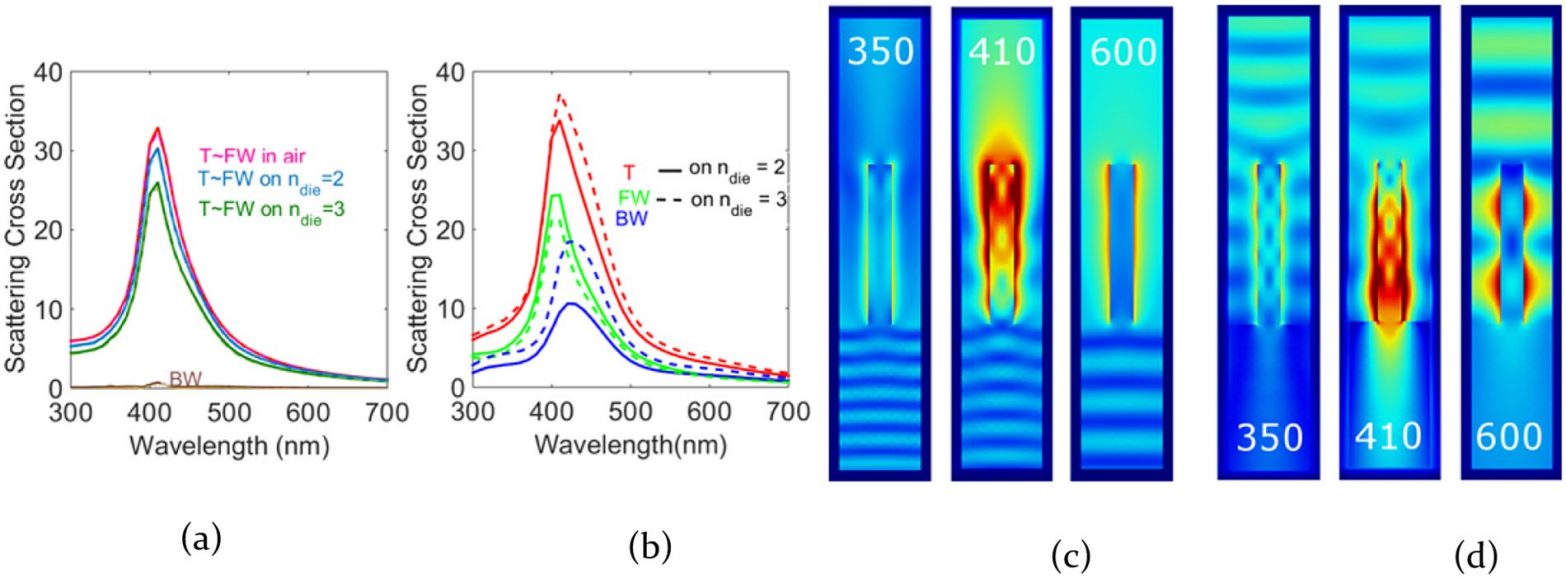
(**a**) Scattering cross section of a ZnO nanorod on various dielectrics with light incident from dielectric (refractive index n_die_) in terms of Total (T), Forward (FW) and Backward (BW) (**b**) Scattering cross section of a ZnO nanorod for light incident directly on the nanorod (**c**) Near field images for light incident on the dielectric with index 2 at wavelengths 350, 410 and 600 nm (**d**) Near field images for light incident in air on the dielectric with index 2 at wavelengths 350, 410 and 600 nm.

Integration of ZnO nanorods for controlling light in optoelectronic devices requires them to control light across material interfaces. It therefore makes it important to observe how nanorods scatter light across an air dielectric interface depending upon the direction of light incidence. An interface results in reflection between the two materials thereby altering the light incident on the rod. The total scattering of the rod as well as the preferential scattering in air and the dielectric would change depending upon the properties of the air dielectric interface^28^. In order to study interface characteristics, we maintained a geometry of radius 60 nm and length 600 nm of a ZnO rod across a dielectric interface with material transitions of refractive index 1 to 2 or 1 to 3 and the rod always being in air. In one case light is incident on the rod from the dielectric and in the other case light is incident on the rod from air. Figure 7(a) and (b) show the scattering spectra of a single nanorod on lossless dielectrics for the two cases, respectively. The interface is used to distinguish between forward and backward scattering contributions depending upon the direction of light incidence. When light is incident on the nanorod through the dielectric (Fig. 7(a)) for an increasing refractive index of the dielectric, the light scattered by the nanorod into air - quantified here by the forward scattering cross section (FW) - decreases. The decrease in scattering can be explained by the evanescent reflection emerging from the interface. Light scattered by the rod into the dielectric - quantified here by the backward scattering cross section (BW) - is negligible and the total scattering cross section (T) is almost approximately equal to the forward scattering cross section (T~FW). As a result of this reflection the total light incident on the nanorod is reduced. It is important to note that the resonance frequency of the rod is unaltered due to the fact that the rod still scatters into air. Figure 7(c) indicates the near field behavior for the case in Fig. 7(a) for response of the nanorod on the dielectric interface (refractive index of dielectric = 2) at wavelengths below, at and above resonance. At resonance forward directivity is maximum. When light is first incident on the nanorod (Fig. 7(b)) there is a substantial increase in the amount of light scattered opposite to the direction of incidence, referred to as backward scattering (BW). The red, green and blue curves show the total, forward and backward scattering cross sections for the air-dielectric interface with dielectric refractive indices 2 (solid curves) and 3 (dashed curves). It can be attributed to the strong near field present in air which then radiates into a strong far field. The backward scattering on the dielectric with refractive index 3 is more than that with index 2 due to the increase in refractive index contrast between air and dielectric which effectively couples more light to the nanorod. Figure 7(d) shows near field distributions of the rod on the dielectric system when light is incident on the rod (corresponding to Fig. 7(b)). At resonance, the directivity into the dielectric is maximum. For both directions of light incidence the overall scattering amplitude remains the same indicating that the nanorod has a strong forward scattering tendency as a result of the aforementioned overlap of leaky modes with dielectric scattering.

### Periodic ZnO Array on Different Air-Dielectric Interfaces

The next step is to examine how the above investigated properties of a single nanorod in air and across an air-dielectric interface translates to a 2D periodic nanorod array and influences the resulting interface reflection and transmission. We maintain a rod array pitch of 200 nm with ZnO rods which have a radius of 60 nm and length 600 nm and vary the air-dielectric interface to have a refractive index range of 1-(1–3 in steps of 0.5) Maintaining a close proximity between the nanorods enables us to observe the enhanced coupling between nanorods for various dielectric interfaces^40^.

**Figure 8:**
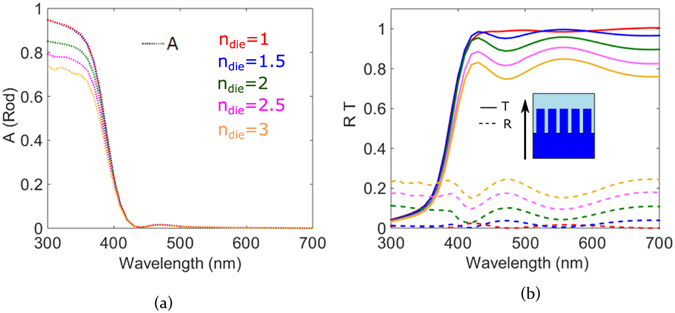
(**a**) Absorption of the ZnO nanorod array on various dielectrics and (**b**) reflection and transmission of various air dielectric interfaces for light incident through the dielectric substrate.

Figure 8(a) indicates the absorption profile of the rod, with light incident through the dielectric substrate for various refractive indices of the dielectric. The individual rod absorbs light in the range between 300 and 400 nm owing to the high value of the imaginary part of the refractive index. Therefore light which is internally confined by it is not able to escape out of the rod via the aforementioned leaky modes and wide angled scattering. As the refractive index of the dielectric is increased, absorption in the rod is gradually reduced, proving that less light is coupled into the rod due to increased reflection as a result of the high index dielectric. The graph in Fig. 8(b) shows the reflection and transmission for the corresponding dielectric values. For the array in air after the ZnO absorption wavelength range, we observe a high transmission of more than 95% and a dip in reflection. If we analyze the case for the dielectric interface between air and material with index 2, we see a peak which lies in the same position as the rod scattering peak at 410 nm. The reflection in this wavelength region significantly decreases; more light is therefore coupled via the forward propagation of the rod. The transmission peak then subsides when the rod is no longer resonant and we observe a corresponding peak in reflection.

For the mid visible wavelengths at 550 nm an additional peak is observed which corresponds to the diffraction of the light transmitted across the structure. This diffraction occurs as a result of the periodicity of the two dimensional rod array. As light is incident from the dielectric on the rods, due to the close proximity between the rods, light propagating in the high index material is strongly coupled out by the grid array via diffraction^41^. This diffraction arises due to the periodicity in the alternating refractive indices of the rod and air separated at subwavelength scales. More light is effectively coupled between the rods as light passes along the rods as a result of this diffraction thereby reducing reflection. A reflection minimum is observed in the wavelength range of the transmission peak. In this configuration, the nanorod array therefore functions like a transmission grating. As the refractive index of the dielectric material is increased, the transmission gradually drops and reflection increases. The dispersion of the transmission curve remains similar for all air-dielectric interfaces.

**Figure 9:**
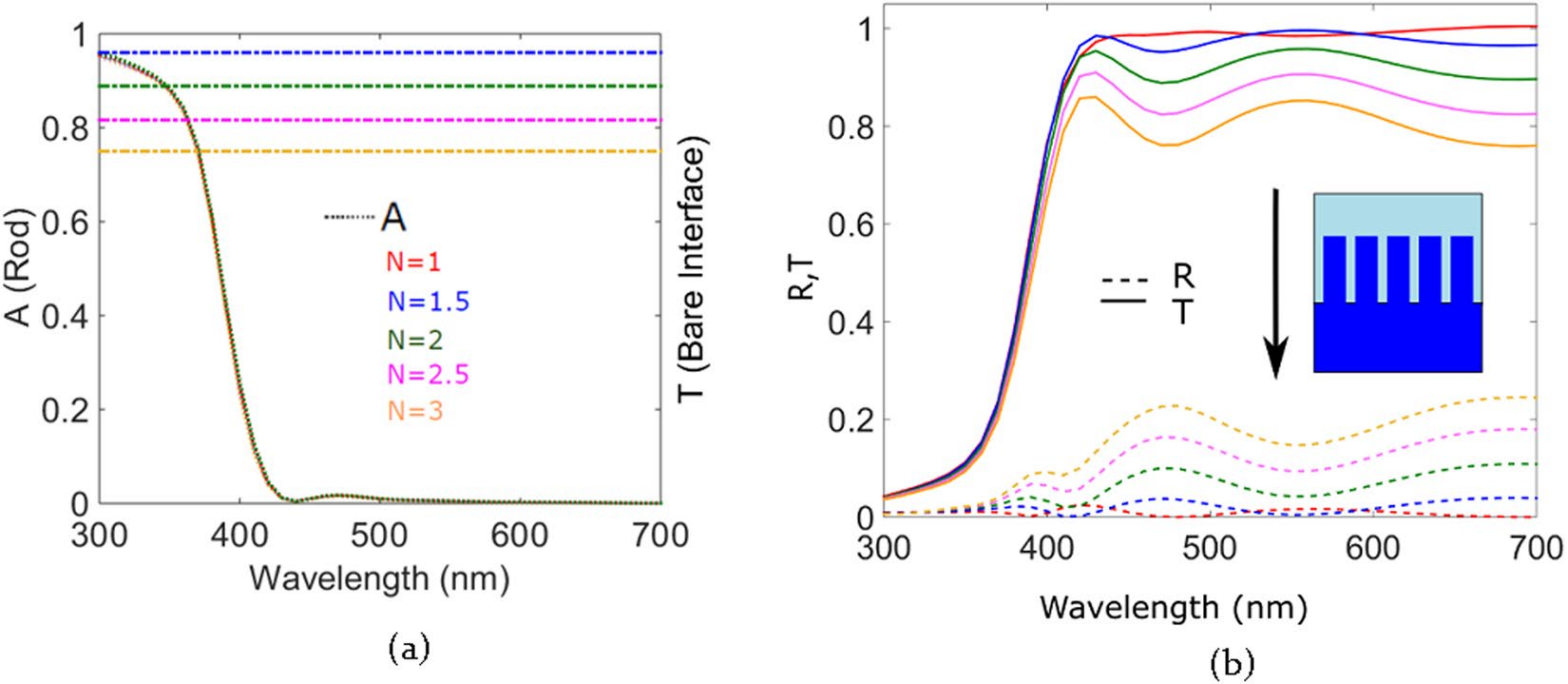
(**a**) Absorption of ZnO nanorod array on various dielectrics and the transmission values for bare interfaces and (**b**) reflection and transmission of various air dielectric interfaces with the nanorod arrays with light incident on the nanorod array.

The key point to note however is that the average transmission is more than what would be observed in case of a bare air-dielectric interface for all refractive index values of the dielectric as shown on the second Y axis seen in Fig. 9(a). The next interesting step is to reverse the direction of light incidence in order to analyze how periodic nanorods couple light to a high index dielectric material. We maintain the same dielectric interface and keep the same rod geometry and pitch between them. Direction of incidence is reversed such that now light is first incident on the rod array.

From the absorption curves in Fig. 9(a), we observe that the rod absorbs uniformly independent of the refractive index of the dielectric below. The amount of light coupled to the rod is not yet influenced by the reflection from the interface, therefore the rod absorbs in the given range. As a result of the high absorption in this short wavelength range, the overall reflection and transmission are low. For longer wavelengths, as a result of the rod scattering, we see a transmission peak which then decays down as the resonance peak of the rod decays giving rise to a reflectance peak ﻿less than 500 nm. Reflection rises just around 400 nm which is due to the back scattering of the rod. Similar grating behavior as previously described is seen here. The average transmission is more than that of the bare interface for this direction of light incidence, too. The nanorods show high forward scattering which results in increased light propagation into the dielectric. Additionally, as the separation between the nanorods is slightly shorter than the wavelength, this results in light being diffracted at the air-dielectric interface. The dielectric has a higher refractive index as compared to air and thereby results in higher number of diffracted modes being coupled into it thereby reducing reflection at the air-dielectric interface^41^. The dip in transmission around 500 nm correspond to the decrease of forward scattering of the rod. Comparing the results for the two directions of light incidence shows that direction of light incidence therefore does not have significant influence on the transmission of the nanorod array on an air-dielectric interface system. It mostly influences the rod absorption and the reflection of the system in the UV and mid visible frequency range. Nanorod array systems therefore can be effectively used to increase coupling light from low index material to high index material and vice versa. This enhanced directivity of nanorods renders them useful in order to increase absorption in a high refractive index absorber such that light is first incident on the rod array system which then couples light into the absorber. In this case it would be interesting to also investigate how the rod array system changes the transmission and reflection spectra on varying the pitch between the rods.

### Pitch Variance and Absorption Enhancement of ZnO Array for a Given Air-Dielectric Interface

For this investigation a constant rod geometry and an air-dielectric interface wherein the dielectric has a constant refractive index of 3 are maintained. The pitch between the ZnO nanorods﻿ of 60 nm radiu﻿s and 600 nm length is then varied between 200–400 nm such that it lies between the UV and visible wavelength ranges. Light is incident on the dielectric via the nanorod array. The thickness of the dielectric is maintained at 600 nm.

**Figure 10:**
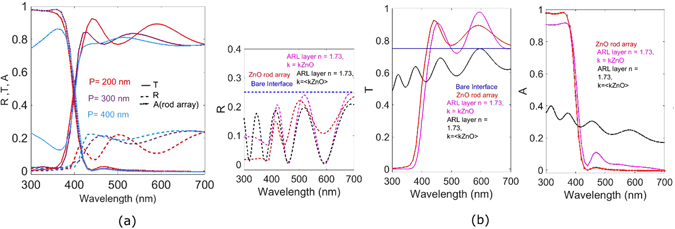
(**a**) Transmission, reflection and rod absorption for different pitches of ZnO nanorod array (radius = 60 nm, length = 200 nm) at the air-dielectric (n = 3) interface (**b**) comparison of reflection, transmission and absorption of the air-dielectric interface in presence of nanorod arrays and planar layers.

For the ease of interpretation, the rod array absorption as well as the reflection and transmission of the total system are plotted in Fig. 10(a). For the shortest pitch (200 nm) in the rod absorption range we observe that transmission across the air-dielectric interface is significantly low. The transmission across the air-dielectric interface increases around the rod resonance in accordance with the trend of transmission as seen in the previous case. Similarly once the rod resonance subsides the transmission decreases resulting in an increase in the reflection of the total system. Further in the visible range, due to increased number of diffraction modes in the dielectric transmission increases. As a counter effect the reflection in the dielectric decreases until the long wavelength range after which reflection rises again. As the pitch is further increased to 300 nm, the transmission peak, previously present at the single rod scattering resonance, now slightly blue shifts. The second transmission peak which is observed as a result of the grating effect shows a similar blue shift and is not as pronounced as compared to the 200 nm pitch. A short pitch translates to higher number of modes in the reciprocal k vector space as compared to a large pitch which supports lower number of modes. Owing to this reduced number of modes, the transmission through the dielectric subsequently reduces for higher pitches^41^. However the nanorod array continues to weakly support diffraction modes, even for the higher pitches, as a result of which the ﻿average transmission through the air-dielectric interface is much greater than for the bare interface. In order to evaluate this increase in transmission due to the periodic nanorod array and the accompanying suppressed reflection, we first compare the reflection due to the nanorod array across the air-dielectric interface to the reflection across the interface in presence of a planar layer with the same thickness as the rod (600 nm) with refractive index as that of an anti-reflection layer (ARL). This helps in comparing the difference between a planar layer and the rod array nanostructure. The real part of the effective refractive index of the anti-reflection layer is the geometric mean of the dielectric and air^42^ which calculates to 1.73. To be able to accurately compare the performance of this ZnO nanostructure, we maintain the absorption coefficient of the ARL having same dispersive properties as ZnO. In this configuration with the anti-reflecting layer, the reflection peaks are slightly red shifted as compared to the reflection peaks with the ZnO nanostructure, (Fig. 10(b) left). At short visible wavelengths reflection of the ARL is significantly higher than that of the nanorod structure due to interference between the two layers. Around 500 nm, the reflection of the nanorod structure rises but it is still lower than that of the ARL. At higher wavelengths, the reflection of the nanorod structure increases, however that of the anti-reflective layer decreases as a result of an increase in its absorption (Fig. 10(b), right). Clearly thus, the planar layer might suppresses reflection but it does in average not transmit more light into the dielectric. Subsequently, if the anti-reflective layer has an absorption coefficient corresponding to average absorption of ZnO (0.02) across the entire wavelength range, reflection is suppressed but more light is absorbed by the material itself across all wavelengths. If we compare the interface transmission with the nanorod array to the planar layers, (Fig. 10(b), middle) then we see increase in the transmission across the specific visible and mid visible wavelength bands. Additionally the rod arrays ensures in minimizing absorption beyond 400 nm. This investigation highlights the superiority of the ZnO nanostructure over planar structures for controlling reflection as well as directing light across the dielectric interface. This felicitates preferential light distribution in terms of offering more spectral flexibility by tuning spectral positions of maximum and minimum reflection and transmission. Nanostructures therefore help in overcoming the limitations of the natural material optical properties primarily exhibited by planar layers. Moreover it is difficult to obtain materials with the exact refractive index matching to the anti-reflection layer. The nanorod periodic array therefore proves to be an effective approach in order to enhance and tailor transmission across the air dielectric interface.

**Figure 11:**
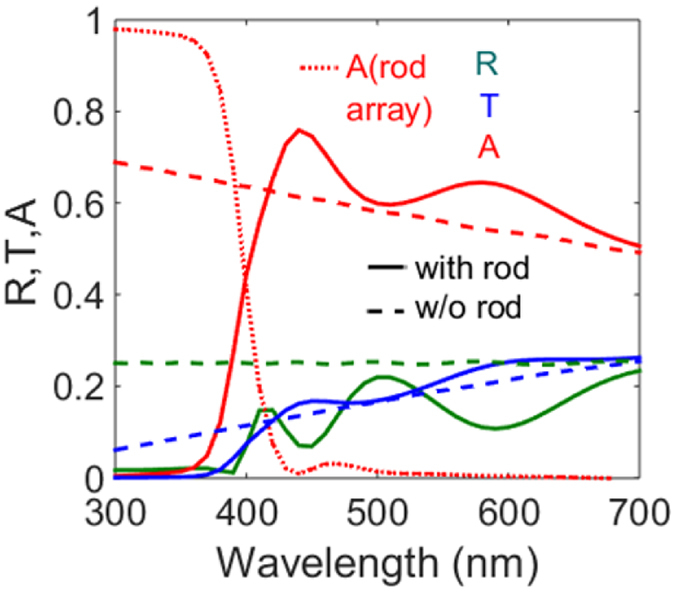
Absorption, reflection and transmission of an air dielectric (n = 3, k = 0.1) interface with periodic ZnO nanorod array (radius = 60 nm, length = 600 nm and pitch = 200 nm) as compared to a bare interface.

To investigate how this array enhanced transmission effectively influences dielectric absorption, in the aforementioned air-dielectric interface with the considered thickness of 600 nm, we introduce a dispersion less absorptive coefficient of 0.1 in the dielectric. For the shortest pitch of 200 nm, we compare the absorption of the dielectric with the rod array against that observed for the bare interface. There is a corresponding peak in absorption at positions where a peak in transmission was absorbed for the aforementioned case of a lossless dielectric. It is evident that absorption now follows the same spectral trend of transmission in the previous case. The dash lined curve in Fig. 11 shows the absorption for the bare dielectric interface and confirms absorption increase in the dielectric due to the rod array. Reflection is also reduced as compared to the bare interface. The rod array thereby enhances absorption in the dielectric by harnessing more modes into the dielectric at mid-visible wavelengths and offering anti-reflection by reducing the mismatch between the refractive indices^43^. Nanorod array systems can therefore be strategically defined for specific configurations of the air dielectric interface in order to couple more light into the dielectric.

### Field Enhancement of a Periodic Array

**Figure 12:**
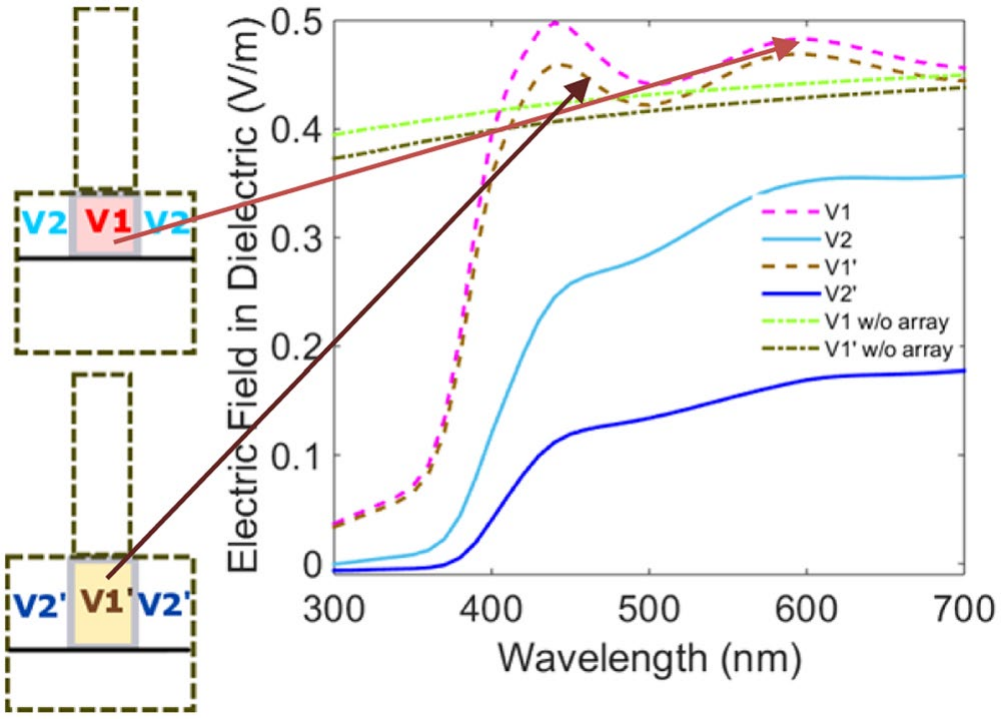
Electric field in regions below and between the rods in the underlying dielectric in comparison with the bare interface. The adjacent configurations shows regions where the field strengths are evaluated for the ZnO rod array (radius 60 nm, length 600 nm and pitch 200 nm). V1 corresponds to the volume inside the dielectric underneath the rod with a depth of 200 nm and V2 corresponds to the volume in the dielectric on either sides of the volume V1 with the same depth. V1’ corresponds to the volume inside the dielectric underneath the rod with a depth of 300 nm and V2’ corresponds to the volume on either side of the volume V1’ with the same depth.

Near field images of the nanorods at resonance indicate a strong field intensity near the rear edge of the rod where light escapes the nanorod. Increased field intensity at this edge of the rod is common for all refractive indices and rod geometries as shown in Figs 2(a–f), 5(b) and (c). A quantification of the increased field strength when the rod array is at the air-dielectric interface for an absorbing dielectric as described in the previous section would illustrate nanorod focusing action. We have already proved an increase in absorption in the dielectric as a combined effect of the nanorod forward scattering and the grating effects of the periodic rod array. In order to evaluate field strengths in the absorbing dielectric just below the rod we evaluate for two different depths (200 nm and 300 nm), the electric field strength in the volume below the rod. The lateral extension of this volume is the same as that of the geometric cross section of the rod and we denote this volume as V1 (depth 200 nm). V2 is the corresponding volume inside the dielectric between two rods in the periodic array at the air-dielectric interface. V1’ and V2’ are similar volumes for the depth of 300 nm as measured from the interface. Figure 12 shows the configuration of the two regions where the fields are calculated. The accompanying graphs indicate the electric field integrated over the aforementioned regions and normalized to their volume. We observe that for the region V1 the electric field inside the dielectric has a spectral pattern similar to that of absorption described in the previous section. The electric field has two distinct peaks corresponding to rod scattering and the periodicity between them. In the region V2 we observe that electric field is significantly lower as compared to region V1. Moreover the two previously observed peaks are absent. A strong electric field in the dielectric directly below the rod is an evidence of focusing action as exhibited by the nanorod array. Conclusions from investigations of nanorod scattering properties and the curve shown in Fig. 12 reveal that such focusing action could be tuned by selecting appropriate rod material and geometric parameters and adjusting the pitch between them in order to arrive at desired spectral properties for any given air-dielectric interface. When we compare this enhancement with respect to a bare interface without the rod array as shown by the light green curve in Fig. 12, the magnitude of electric field inside the dielectric is low. Reflections arising out of the bare interface result in a low electric field strength inside the dielectric.

On an absolute scale, the increase in enhancement might not appear large however the point to note is that this enhancement is a local enhancement, calculated in the immediate vicinity of the nanorod. A local increase in field strength implies that nanorod arrays could be used to focus light in a very small region in the immediate vicinity of the nanorod. If we move slightly away from the interface into regions V1’ and V2’ we observe that the electric field undern﻿eath the rod follows the same spectral dispersion (dark brown curve) but has a lower amplitude. It is clearly greater than the bare interface field strength, indicating that field u﻿﻿nderneath the rod is enhanced locally by the rod array and decays as the distance from the rod array increases. The field strength in regions V2 and V2’ is less than that in regions V1 and V1’ thereby confirming strong focusing properties of the rod array. To conclude, for values of V1 in close proximity of the rod, the effective electric field value is enhanced. This behavior opens avenues for applications such as solar micro-concentrators and small emitters which require strong field strength confined over a small area. Spectral tunability offered by nanorod arrays increases the spread of applications which can utilize focusing properties of nanorod arrays.

## Conclusion

In this work we started with a single dielectric nanorod and illustrated how it interacts with light for a given material and geometry. This highlights strong light directivity exhibited by nanorods. Our investigations reveal that a single nanorod under axial illumination exhibits leaky mode enhanced dipolar scattering for moderate aspect ratios. A strong near field enhancement is seen as a result of the coupling between leaky modes and scattering. Variations in geometry and material strongly influence scattering amplitudes as well as spectral positions of resonances and can be used to design rods to arrive at the desired spectral properties offering tremendous wavelength flexibility to cater to optoelectronic applications like detectors, sensors and solar energy devices. Calculations elaborating the influence of the surrounding medium refractive index help in determining the effective light scattering for a given dielectric surrounding. Optical properties of a periodic nanorod array across different air-dielectric interfaces indicate the influence of the nanorod on the spectral transmission and reflections which can be controlled by selecting nanorods of suitable materials and geometry^44^. Differences in spectral properties for different directions of light incidence show how these periodic systems can reveal varying reflection in the UV absorption range of the nanorod for air dielectric interfaces and can help in increasing transmission by 20% relative. We evaluate how varying pitches between the nanorods influences for a lossless air dielectric interface the spectral transmission and reflection along with their magnitudes. We evaluate this enhancement with respect to anti-reflection layers of different loss coefficients to conclude that nanorod array structure across the considered spectrum have better performance as compared to the anti-reflection layer. Nanorod arrays have been shown to provide better spectral control of the reflection and transmission spectra as against conventional planar anti-reflection layers. The configuration for which maximum transmission is observed is then investigated with an absorptive dielectric which proves to enhance its absorption as compared to a bare air dielectric interface without a rod array. We also evaluate the localized high field enhancement in a small volume at the end facet of the nanorod and imply the deployment for light focusing applications. We therefore demonstrate the unique properties of dielectric nanorods with low absorption in short bandwidth for light coupling across material interfaces and preferential field localization as per requirement.

## Methods

For our investigations we have used Comsol Multiphysics^44^ to simulate different configurations of single and periodic nanorod arrays. We verified the results using JCMWave suite^45^. The computation is performed using the finite element method in a two-step process for single nanorods by first calculating the background field for the air substrate interface without the rod and then evaluating the scattered field of the rod on substrate interface. Perfectly matched layers are used at the simulation boundaries to eliminate unrealistic reflections that add to the scattered field solution. Scattering cross sections are calculated with the aid of three Poynting vector components. The three components are integrated across the computational domain boundaries. The total integrated Poynting vector is then normalized with respect to the input power. The input power is calculated for the computational domain and all optical materials taking into consideration an input electric field of 1 V/m polarized along the cross section of the rod. In all calculations, light is always incident parallel to the rod axis, as a result of the rod symmetry the field, the aforementioned results would be indifferent towards polarisation of the incident field. The scattering cross sections are normalized with respect to the effective area seen by the incident light which corresponds to the geometrical cross section of the rod. For periodic simulations, a one-step calculation is performed with periodic boundaries and maintaining the PML above and below the excitation and reception port. Reflection and transmission are calculated by the Poynting vector flux integrals on the excitation and reception ports respectively. Rod absorption is calculated by integrating the electric field energy density over the rod volume. In order to calculate the absorption of the dielectric in the air-dielectric interfaces, we use the same procedure of the electric energy density integral over the volume of the dielectric.

## Electronic supplementary material

Supplementary Information
