# Supplementary material for: Dielectric Nanorod Scattering and its Influence on Material Interfaces
Source: Sci Rep. 2017 Jun 27;7:4311. doi: 10.1038/s41598-017-03721-w (PMC5487353; doi:10.1038/s41598-017-03721-w)
Supplement: Supplementary file 1 — Supplementary Information [file 41598_2017_3721_MOESM1_ESM.pdf]

Dielectric Nanorod Scattering and its Influence on Material Interfaces

Gauri M. Mangalgiri[1], Phillip Manley[1], Wiebke Riedel[2] and Martina Schmid[1, 3]

[1]Nanooptische Konzepte für die PV, Helmholtz Zentrum Berlin für Materialien und Energie, 14109 Berlin, Germany, [2] Freie Universität Berlin, Department of Chemistry, 14195, Berlin, Germany ,[3] Freie Universität Berlin, Department of Physics, 14195, Berlin, Germany

BG Keywords: Dielectric resonances, light coupling, light enhancement, interfaces, ZnO nanorods

SUPPLEMENTARY INFORMATION

Pertaining to Figure 2 of the paper.

In order to affirm the fact that dielectric nanorods from materials like ZnO exhibit leaky modes and thereby give rise to strong Poynting vector fluxes we evaluate the near-fields obtained from the FEM solutions at the wavelength of 410 nm. At this wavelength the scattering cross section is maximum. Figure 13 shows the y-component of the electric field along the nanorod length in the plane of polarisation. The incident port location is at z= 50 nm and the rod is at z = 600 nm and spans upto z= 1200 nm. Light is first incident on the rod at z = 600 nm. As light propagates along the length of the rod, the absolute value of the y-component of the electric field increases for every point of maxima and minima, indicating oscillation along the y-direction in the transverse plane Since the imaginary part of the rod refractive index is almost zero, light travels in a high index material with maximum amplitude (along z) which is spread in the transverse direction (y). The near field images shown in the transverse (xy) and longitudinal (yz) directions in figure 2 indicate this behavior. After the rod length, the field amplitude monotonically decreases.

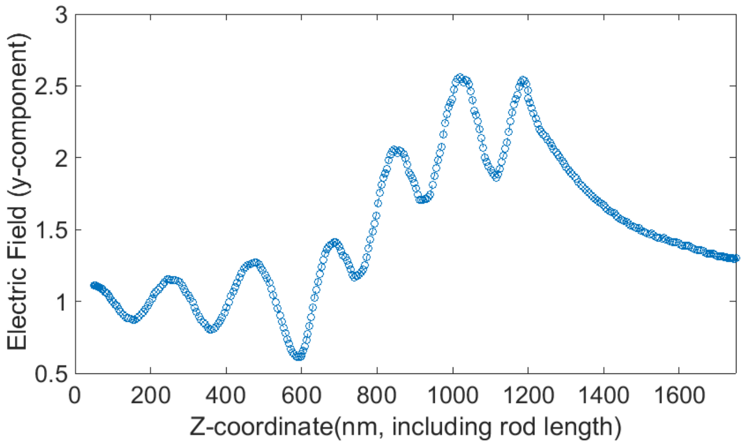

Figure 13: Y-component of the electric field plotted along the rod length at the scattering peak of 410 nm for a ZnO nanorod in air .
